# The Power, Wisdom, and Goodness of God

**Published:** 1839-01

**Authors:** 


Art. V.—*The Power, Wisdom, and Goodness of God, as displayed in the Animal Creation: showing the remarkable Agreement between this Department of Nature and Revelation. In a Series of Letters.* By C. Burnett, m.r.c.s.—*London,* 1838. 8vo. pp. 530.

Although this book is written with a very laudable object, and with a pleasant freedom of style which will render it acceptable to the general reader, we cannot perceive any such novelty, either in its plan or style of execution, as need have demanded its publication. We have scarcely noticed a single fact which is not already familiar to the readers of the various widely-diffused popular essays on this subject: indeed, a very large part of the volume is made up of quotations from the Bridgewater Treatises and other similar productions. We have often wished that some one competent to the task would bring together, in a compendious form, the reasonings scattered through that valuable series, supplying many links which the unphilosophical partition of the subject has left unfilled, and suppressing the many repetitions which the peruser of the whole encounters. Such, we at first hoped, was the object of the author of the present volume, in regard to the animal creation at least; but, though his avowed scope is so wide, the number of subjects he has embraced is really very small. The first two letters are designed to overthrow the received doctrines of geology, and to establish the propriety of the literal interpretation of the Mosaic account of the creation. We shall not here enter upon the discussion of this *quæstio vexata,* since it is out of our province; but we would recommend to Mr. Burnett and his friends the perusal of a work which we have formerly noticed with approbation, and which emanates from a source that at once protects it from the imputation of regardlessness of the value of the sacred records, and establishes its claim to impartial attention: we allude to the essay on the "Connexion between natural and revealed Truth," by the Rev. Professor Powell, of Oxford. The view which is there given of the present state of geological science, in reference to what is regarded by many as revealed truth, is, we have reason to know, adopted by some of the highest authorities on the subject, and is much better worth Mr. Burnett's attention than the hypothesis which rests upon the extension of the "day," which is now abandoned by most geologists of eminence in this country. We are sorry to be obliged to speak with disapprobation of many other parts of Mr. Burnett's treatise, which are likely to mislead the general reader. For instance, he speaks (p. 151) of heat as "the prime agent in carrying on the functions of every organ of the body;" and then tells us that "this power is not inherent in itself, but is communicated to it by the agency of another principle, called electricity, which is rendered subservient to the purposes of animal existence by means of a particular set of organs, whose function it is to transmit it." From this it follows that "heat and electricity, by their joint influence, produce the phenomena of secretion." Further on, we find it stated that "it is quite evident that, in the present state of science, our knowledge of the principle of life is limited to the fact that atmospheric air, by means of the stimulating power of its oxygen, propels the blood through the lungs, and thus gives rise to all the vital phenomena." (p. 162.) We are really sorry to be obliged thus to put our readers on their guard against the doctrines laid down in this work, because, as we have already said, it is written with a laudable object, and conveys much useful information in a generally acceptable style; and we shall therefore close our notice with what may be regarded as favorable specimens of the author's style in description and in deduction.

"Perhaps the most singular adaptation of the eye to the two media of air and water is seen in the anableps, an animal which inhabits the rivers of Guiana. The orbit or socket of the eyeball extends so far above the head, that the eye, as the animal swims near the surface, is partly in and partly out of the water; and all its internal parts correspond with this curious external conformation. The iris being partially divided into an upper and a lower portion, there are consequently two distinct pupils; the cornea consists of two globes, an upper and a lower one, attached together but divided by a dark band; the anterior lobe, which the animal uses out of the water, is in all respects like the eye of terrestrial animals, adapted to refract rays transmitted through the rare medium of air; the inferior one, which is always under the water, like the eye of aquatic animals, is adapted to refract light transmitted through the denser medium of water. So that the refracting power of the upper globe is less than that of the lower." (p. 410.)

"Animals, in their natural language of inarticulate sounds and gestures, find a facility of expression amply sufficient to serve the limited purposes of their creation; but man, whose race is spread over every climate of the globe, and whose progression depends upon the intellectual exertions which he is able to make, finds in artificial language the only means of communication commensurate with his wants and worthy of his exalted nature. Speech, then, loudly proclaims the fact that man possesses the superior powers of reason and judgment, which involve the high moral responsibility that attaches to his existence here; and hence, by a chain of reasoning which forces itself upon our minds, we are led to the conviction that he has been placed on the earth by a wise and intelligent Being, for objects and purposes which, bearing no relation to anything in the present state of existence, silently but awfully remind him that he is on his passage to another, and, as he is taught to believe, a happier and a higher sphere." (p. 212.)

